# Histological Examination of Horse Chestnut Infection by *Pseudomonas syringae* pv. *aesculi* and Non-Destructive Heat Treatment to Stop Disease Progression

**DOI:** 10.1371/journal.pone.0039604

**Published:** 2012-07-09

**Authors:** Jeroen de Keijzer, Lambertus A. M. van den Broek, Tijs Ketelaar, André A. M. van Lammeren

**Affiliations:** 1 Laboratory of Cell Biology, Wageningen University and Research Centre, Wageningen, The Netherlands; 2 Wageningen UR, Food & Biobased Research, Wageningen, The Netherlands; Nanjing Agricultural University, China

## Abstract

Since its emergence in Northwest Europe as a pathogen that infects trunks and branches of *Aesculus* spp. (the horse chestnuts) approximately one decade ago, *Pseudomonas syringae* pv. *aesculi* has rapidly established itself as major threat to these trees. Infected trees exhibit extensive necrosis of phloem and cambium, which can ultimately lead to dieback. The events after host entry leading to extensive necrosis are not well documented. In this work, the histopathology of this interaction is investigated and heat-treatment is explored as method to eradicate bacteria associated with established infections. The early wound-repair responses of *A. hippocastanum*, both in absence and presence of *P. s.* pv. *aesculi*, included cell wall lignification by a distinct layer of phloem and cortex parenchyma cells. The same cells also deposited suberin lamellae later on, suggesting this layer functions in compartmentalizing healthy from disrupted tissues. However, monitoring bacterial ingress, its construction appeared inadequate to constrain pathogen spread. Microscopic evaluation of bacterial dispersal *in situ* using immunolabelling and GFP-tagging of *P. s.* pv. *aesculi*, revealed two discriminative types of bacterial colonization. The forefront of lesions was found to contain densely packed bacteria, while necrotic areas housed bacterial aggregates with scattered individuals embedded in an extracellular matrix of bacterial origin containing alginate. The endophytic localization and ability of *P. s.* pv *aesculi* to create a protective matrix render it poorly accessible for control agents. To circumvent this, a method based on selective bacterial lethality at 39°C was conceived and successfully tested on *A. hippocastanum* saplings, providing proof of concept for controlling this disease by heat-treatment. This may be applicable for curing other tree cankers, caused by related phytopathogens.

## Introduction

**Figure 1:**
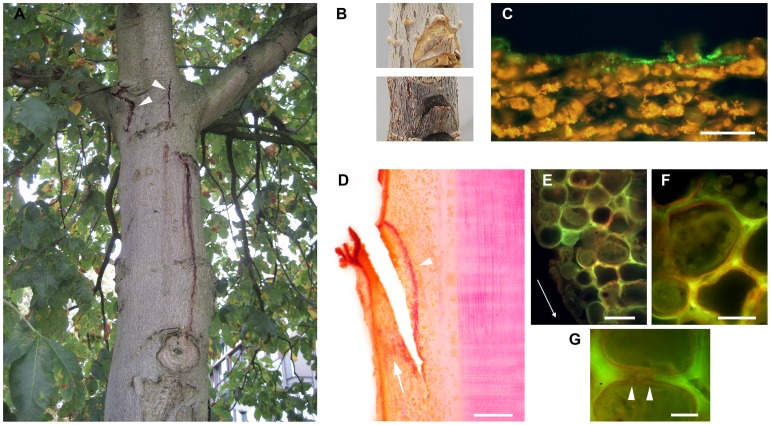
Bleeding disease of horse chestnut and biogenesis of a structural barrier after wounding. (A) Typical symptoms of *P. syringae* pv. *aesculi* associated bleeding disease observed on the trunk of an *A. carnea* tree, including bleeding of amber coloured sap and cracking of the bark (arrowheads). Photo taken in Sept. 2008 at N51° 57′ 26″; E5° 34′ 10″. (B) Appearance of surface wounds on *A. hippocastanum* seedlings mock-inoculated (top panel) or inoculated with *P. s.* pv. *aesculi* PD5126 (bottom panel) after 3 months. While the seedling bark recovers when mock-inoculated by regeneration of periderm, the wounded tissue appears blackened and sunken when inoculated with *P. s.* pv. *aesculi*. (C) Immunofluorescent labelling of a transverse section of the wound area sampled directly after inoculation of *P. s.* pv. *aesculi* PD4818 (t = 0) indicates that the bacteria (green) only colonize the outermost cell layer after inoculation. The scale bar indicates 100 µm. (D) A longitudinal section of a mock-inoculated wound sampled after 6 days with phloroglucinol-HCl stained lignin (red/purple). A barrier zone composed of several lignified parenchyma cells wide is visible along the wound (arrowhead) along with a broad layer of diffusely lignified cells in the disjointed part (arrow). The scale bar indicates 0.5 mm. (E) Fluorescence microscopic observation on a longitudinal section of a wound sampled 8 days after PD4818 inoculation stained for waxes using Sudan IV. The cells that exhibit lignification, as seen by the yellow/green autofluorescence, also show Sudan IV stained suberin lamellae that appear in dim red. The arrow reflects the direction of the original inoculation cut. The scale bar indicates 50 µm. (F) Detail of E showing the even deposition of suberin around the plant protoplasts. The scale bar indicates 25 µm. (G) Continuous deposition of suberin by two cells at pit fields (arrowheads). The scale bar indicates 10 µm.

Since the last decade necrotic lesions/cankers with gummy exudations are observed on stems and branches of trees in the *Aesculus* genus, most notably the white horse chestnut (*A. hippocastanum*). These symptoms are found in Northwest Europe, with reports from Belgium [1], Germany [2], the United Kingdom [3] and The Netherlands [4]. The disease is severe, with a large part of the population exhibiting the typical bleeding symptoms [4], [5]. It manifests itself on trunks and branches where it causes necrosis of bark tissues and bleeding of an amber coloured sap, which turns black in later stages (Fig. 1a). Often the vascular cambium becomes necrotic, leading to irregular secondary growth. When necrosis of the phloem is extensive, tree vitality is reduced and girdling lesions ultimately lead to dieback.

While *Phytophthora* species are known to cause bleeding symptoms on horse chestnut [6], this recent outbreak was consistently found to be associated with the Gram-negative bacterium *Pseudomonas syringae* [1]–[4]. Intriguingly, the new European isolates were found to show a high degree of genetic similarity to an earlier described Indian *P. syringae* pathovar that causes leaf spots on *A. indica* [7], and were therefore accommodated in the same pathovar *aesculi* [2], [3], [8]. Indeed the sudden virulence of this horse chestnut pathogen is almost certainly due to its recent arrival in Europe, probably being introduced from India some time shortly preceding the epidemic in Northern Europe [8].

European *P. syringae* pv. *aesculi* is found as an epiphyte on leaves, flowers and fruits of the host tree [9]. The bacterium can adopt an endophytic, pathogenic lifestyle in the presence of small wounds, for example leaf scars or superficial injuries caused by human activities [10], [11]. Further potential entry points for the bacterium have been shown to include lenticels [11], although spray-inoculation of these sites has been reported unsuccessful, even under lenticel opening conditions [10]. Once an infection is established, it may spread tangentially (in a diffuse manner) or in a predominantly longitudinal direction [10], [11]. From the centre of old lesions no or little bacteria can be identified, in contrast to their edges and fresh lesions [5]. This hints at active colonization of new host tissues, but exact routes of bacterial dispersion and sites of multiplication are not known.

Wounded plant tissue can lead to desiccation and forms a potential entry point for pathogens. Therefore, it is of importance for plants to separate healthy from disrupted tissues. Generally, wounds or infection sites in tree barks can be sealed off by *de novo* periderm (consisting of phellem, phellogen and phelloderm) formation, which is considered a non-specific defence response [12]. Formation of a new periderm layer near infection courts in *Aesculus* bark has been observed, but appeared ineffective in preventing (further) bacterial invasion [4], [11]. However, before the development of a new periderm, cell wall reinforcing components are deposited by existing cells near wounds in many tree species [13]–[16]. In this context, lignin and suberin are found as widespread components of initial wound-repair and may function in preventing water loss and further tissue collapse [13]. For horse chestnut the timing and nature of such structural cell wall reinforcements in a wound boundary zone have not been described and its possible role in preventing the development of bleeding disease is unknown.

Understanding of bacterial behaviour and localization within horse chestnut tissues might provide insights in the sudden virulence of this novel horse chestnut pathogen and may be of value for comprehension of the infection process of other, related pathogens. In this study the early infection process of *P. syringae* pv. *aesculi* is visualized in artificially inoculated *A. hippocastanum* seedlings using immunohistochemistry and constitutive, plasmid-borne GFP expression. The early structural defence response is characterised using various histochemical stains, and its relation to bacterial spread is evaluated. Lesion edges were observed to discern potential routes of bacterial migration through host tissues. Additionally, an approach encompassing selective, heat-based control of the causal bacteria is explored.

## Results

To investigate the early events after *P. s.* pv. *aesculi* comes in contact with internal host tissues, bacteria were applied to small wounds inflicted on the stems of *A. hippocastanum* seedlings. The method of wounding did not cause irrecoverable damage, since mock inoculated wounds showed generation of a new periderm confluent with the existing one within 3 months (Fig. 1B upper panel). Inoculated wounds did not exhibit such a recovery (Fig. 1B lower panel). Microscopic analysis of the inoculation site directly after application of the bacteria (t = 0) revealed that the pathogen only colonized the ruptured cells on the surface of the wound (Fig. 1C), assuring that the inoculation method itself did not bring the bacteria in deeper tissues.

### Lignin and Suberin are Deposited Near the Wound Boundary

When inoculation sites were microscopically observed, an autofluorescent lining that followed the borders of the wound was consistently discerned from the second or third day after the moment of inoculation and onwards, in both mock and *P. s.* pv. *aesculi* inoculated stems. The autofluorescence was confined to the cell walls of a continuous layer of parenchyma cells, several cells wide. Sections of sampled time points were stained with histochemical dyes to define the cell wall components that make up the observed lining near the wound boundary. Using phloroglucinol-HCl staining it was determined that the localization and timing of the observed layer of autofluorescent cells corresponded with distinct appearance of lignin (Fig. 1D). The distribution of lignin was restricted to several cells wide for the inwards flank of the wound (Fig. 1D, arrowhead) and more diffusely spread in the disjointed part of the wound (Fig. 1D, arrow). Often, the parenchyma cells that participated in lignin impregnation were located at a variable distance from the wound surface, suggesting a signal was relayed from the wound surface to these cells. Altogether, these findings indicate that lignin is a major component of the early wound repair responses in *A. hippocastanum* bark.

Besides lignin another histological compound with autofluorescent properties that could potentially be of importance in the barrier lining the wound is suberin [13], [14], [17]. To simultaneously visualize suberin and lignin, Sudan IV stained sections were observed under fluorescence conditions whereupon suberin lamellae appeared in dim red, while lignin autofluorescence was unaltered (See materials and methods). From the sixth day after inoculation and onwards suberin was found as a lamella against the cell wall of the cells that exhibited lignification earlier (Fig. 1E/F). The deposition was uniform and did not occlude pit fields (Fig. 1G), supporting that the lining of lignified and suberized cells around the wound perimeter functions in sealing off the wound from healthy tissues. No comprehensive differences regarding the timing and extent of suberin deposition were detectable between *P. syringae* and mock inoculated wounds. This hints that the observed layer of cells with lignified and suberized walls is an intrinsic response of *A. hippocastanum* to wounding, which also takes place in the presence of *P. s.* pv. *aesculi*.

### Initial Plant Responses do not Prevent Bacterial Spread

**Figure 2:**
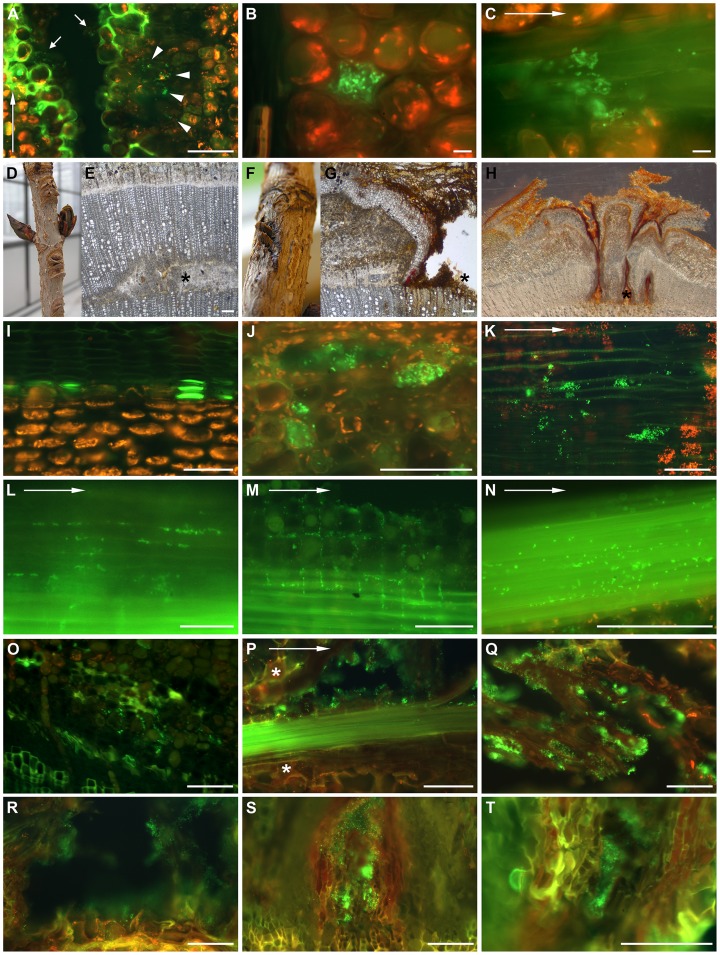
The infection process of *P. syringae* pv. *aesculi* and bacterial behaviour *in planta*. The infection process up to 18 weeks after inoculation visualized by immunocytochemically labelling of *P. syringae* pv. *aesculi*. Arrows in A, C, K, L, M, N and P indicate the longitudinal direction. Scale bars indicate 100 µm in all micrographs except B and C where 10 µm is indicated. (A) Six days after inoculation, when the lining of lignified and suberized cells is prominent, clusters of bacteria are observed in deeper parenchyma tissues (arrowheads) by immunolabelling. Furthermore, bacteria remain in the original inoculation cut (arrows). The image shown is taken from a longitudinal section. (B) A bacterial accumulation in a cortex parenchyma cell observed in a longitudinal section of an infection site sampled 14 days after inoculation. Besides the large cluster, several individual bacteria are scattered in intercellular spaces. (C) A longitudinal section of phloem tissue 6 days post inoculation showing the decoration of bark fibres by *P. s.* pv. *aesculi* seemingly following the vertical orientation of these elements. (D, E) Close-up and micrograph of transversal section of a mock-inoculated site on a horse chestnut plant 7 weeks after inoculation. The asterisk points to site of wounding during inoculation. (F, G, H) Close up and transversal sections of inoculation sites 15 weeks after inoculation. Note partially restored development of secondary xylem despite infection. Asterisks point to site of wounding during inoculation. (I, J) Transversal sections of cortex in mock-inoculated (I) and infected (J) horse chestnut 12 days after inoculation. Note ablated plant cells filled with bacteria in J. (K) Clusters of bacteria in a longitudinal section of the xylem close to the inoculation site, 4 weeks after inoculation. (L–N) Longitudinal sections of the cambium region with bacteria in intercellular spaces of phloem parenchyma (L), ray parenchyma (M) and along phloem fibres (N), 3 weeks after inoculation. (O) *P. s.* pv. *aesculi* in intercellular spaces observed in a transversal section of the cambial zone at 4 weeks after inoculation. (P) Bacteria decorating a bundle of phloem fibres which is surrounded by newly formed periderm (*) and degenerating parenchyma 4 weeks after inoculation. (Q) Bacteria colonizing necrotic bark tissue at 10 weeks after inoculation. (R-T) Transversal sections of enclosures surrounded by periderm with bacteria *ex planta* at 4, 10 and 18 weeks after inoculation, respectively.

The effectiveness of the cellular response in restraining pathogen invasion after wounding can be assessed when related to observations on bacterial proliferation. Using immunolabelling, the spread of inoculated *P. s.* pv. *aesculi* was monitored. During the first 2 days after inoculation, the bacteria were still mainly localized on the wound surface, with some individual bacteria present several cell layers deeper (data not shown). At the fourth day after inoculation several scattered bacteria were found behind the developing barrier zone. These first signs of bacterial ingress were clearly discernible in samples taken 6 days post inoculation, in which small clusters of bacterial cells were found in intercellular spaces of parenchyma cells, evidently inward from the lignified and suberized lining (Fig. 2A, arrowheads). Besides the colonization of living tissues, bacterial clusters remained in the wound fissure (Fig. 2A, arrows).

During the days after the first bacterial clusters were observed behind the histological barrier, the first disease effects were visible on the cellular level. Individual parenchyma cells were found to be ablated and replaced by a large cluster of bacteria (Fig. 2B). Other, solitary bacteria were often found in the intercellular spaces of surrounding cells. One week post inoculation, bacteria were also found in the cambial region, although signs of destructive nature were not yet recorded in this tissue. Interestingly, the bacteria were found distributed along the sclerenchyma fibres of the phloem adjacent to the wound surface (Fig. 2C).

### Bacterial Spread on the Long Term

Bacterial behaviour was further monitored up to 18 weeks after inoculation. Whereas mock inoculated plants exhibited wound healing and continued secondary growth at the inoculation site over a period of 15 weeks, infected plants showed reduced secondary growth (Fig. 2D–H) and necrosis that expanded in longitudinal direction. Twelve days after inoculation, clusters of bacteria were still found in ablated cells in the living cortex (cf Fig. 2 I and J) and at 2 and 3 weeks post inoculation, bacteria were observed in the phloem close to the cambium and incidentally in the xylem, although exclusively in the vicinity of the inoculation site (Fig. 2K).

Three to four weeks after inoculation many bacteria were observed in the intercellular space of phloem parenchyma (Fig. 2L), ray parenchyma (Fig. 2M), around phloem fibres (Fig. 2N), and between phloem cells close to the cambium region (Fig. 2O). As such, intercellular space might facilitate the longitudinal and radial spread of bacteria. As a reaction upon infection, phloem fibres decorated with bacteria were often surrounded by periderm (Fig. 2P).

In samples taken 10 and 18 weeks after inoculation, bacteria were found at the edges of the expanding lesions, *ex planta* in degenerated bark (Fig. 2Q), at the open surface of the wounded area (Fig. 2R), and in enclosures of necrotic tissue adjacent to the cambium but surrounded by periderm (Fig. 2S–T). Thus, bacteria were found in both degenerated as well as still degenerating bark tissue during the whole period of investigation.

### Expression of GFP in the *aesculi* Pathovar

To be able to study the bacteria *in planta* more accurately and uncover possible dynamic behaviour, an approach to constitutively express GFP in *P. s.* pv. *aesculi* was undertaken. To facilitate GFP expression in plant-associated, Gram-negative bacteria Bloemberg *et al.* [18] constructed plasmid pMP4655, which features the eGFP-encoding gene driven by the *lac* promoter (Table 1). Replication functions of this plasmid are derived from the *Pseudomonas* stable plasmid pVS1, known for high stability in absence of selection pressure [19], which is required when bacteria are associated with their host.

**Table 1:** Bacterial strains and plasmids used in this study.

Strain or plasmid	Relevant characteristics^a^	Reference or source
*P. syringae* pv. *aesculi*		
PD4818	*A. hippocastanum* isolate, Winssen, the Netherlands, 2004, Cb^R^	Plantenziektenkundige Dienst, Wageningen, the Netherlands
PD5126	*A. hippocastanum* isolate, the Netherlands, 2005, Cb^R^	Plant Research International, Wageningen, the Netherlands
*E. coli*		
DH5α	F^−^, φ*80*Δ*lacZM15*, *recA1*, *endA1*, *hsdR17*, *phoA*, *supE44*, *gyrA96*,*relA1*, λ^−^	[48]
GM119	F^−^, *dam* ^−^, *dcm* ^−^, *metB1*, *lacY1*, *galK2*, *galT22*, *tonA31*, *tsx-78*,*supE44*, *mtl-1*	Dr. M. G. Marinus (University of Massachusetts medical school)
Cel40	Tra^+^ (pRK2013), Km^R^	E. L. Lagendijk (Molecular Microbiology Leiden University)
plasmids		
pMP4655	oriBBR1, oriVS1, oriT (p15A), *lac*::*eGPF*, Tc^R^	[18], [19]
pRK2013	oriColE1, Tra (RK2), Km^R^	[49]

a Abbreviations used: Cb, carbenicillin; Km, Kanamycin; Tc, Tetracycline; Tra, conjugal transfer functions.

The two *P. s.* pv. *aesculi* strains used in this study (Table 1) appeared to be extremely refractory to transformation methods promoting physical displacement of plasmid DNA across the cell membrane (i.e. electroporation and heat-shock methods; data not shown). Also, using plasmid DNA prepared from methylation deficient *E. coli* strain GM119 (Table 1) did not improve transformation efficiency, as was described for other *P. syringae* strains [20]. A triparental conjugation protocol was therefore employed, using carbenicillin as selective agent to favour *P. syringae* growth after conjugation (See materials and methods for details).

**Figure 3:**
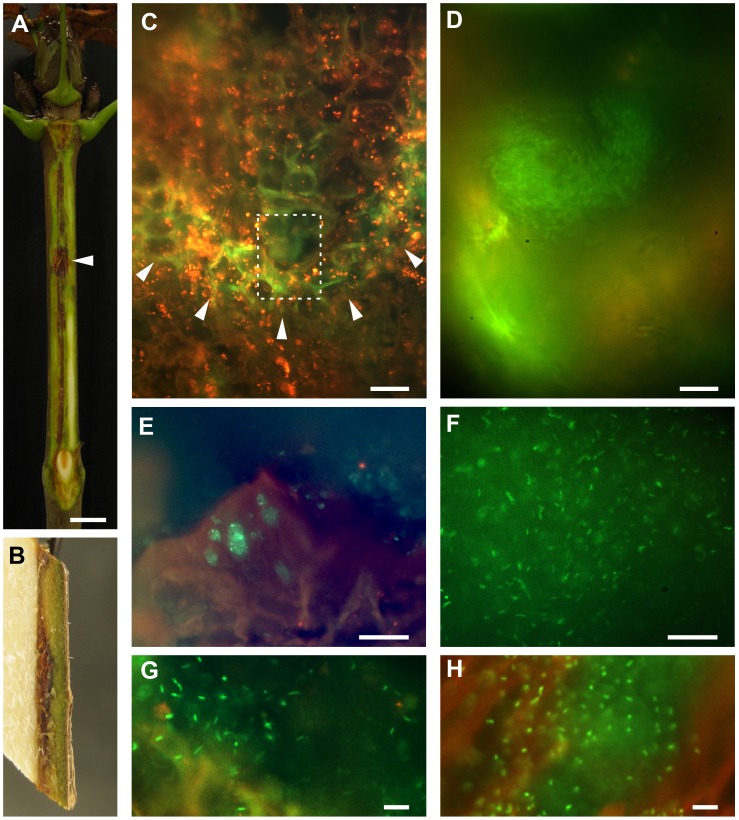
Behaviour of GFP-expressing *P. syringae in planta.* (A) An *A. hippocastanum* seedling inoculated with strain PD4818-pMP4655 photographed after 2 months shows vertical lesion expansion (up to 6 cm in length) and tissue necrosis after removal of the outer tissues of the bark. The inoculation site is indicated with an arrowhead. Note the bending of the infection around the lower node. The scale bar indicates 1 cm. (B) Side view of a longitudinally split piece of stem from a PD4818-pMP4655 inoculated seedling removed 1–2 cm from the inoculation site 4 weeks after inoculation. Necrosis and discolouration is visible in the cambial and phloem areas. (C) A longitudinal section of an infection border in bark tissue after inoculation with PD4818-pMP4655 sampled 4 weeks after inoculation. A parabolic shaped zone with elevated cell wall autofluorescence is discernible (arrowheads) with a *P. s.* pv. *aesculi* colony located in the tip of the infected zone, at about 1 cm away from the inoculation site. The boxed area is shown in detail in D. The scale bar indicates 25 µm. (D) Numerous bacteria are clustered near the forefront of an advancing lesion in parenchyma 4 weeks after inoculation. The bacteria observed here are relatively small and densely packed. The scale bar indicates 10 µm. (E) Within the necrotic area near the original site of inoculation several clusters of bacteria are found that occupy the cavities left by dead cells. Image taken from a longitudinal section of a 6 week old infection. The scale bar indicates 25 µm. (F) A large aggregate of PD4818-pMP4655 located in necrotic tissue of a 4 week old lesion. Individual bacteria were spaced apart and did not undergo any type of motility. The scale bar indicates 25 µm. (G) Detail of a PD4818-pMP4655 cluster in necrotic tissue of a 4 week old infection, emphasizing the spacing between individual bacteria. The scale bar indicates 10 µm. (H) Immunodetection of wild-type PD4818 in the necrotic tissue of a 10 week old infection showing a similar spatial arrangement of bacteria to that shown in G. The scale bar indicates 10 µm.

Transconjugants were obtained for both *P. s.* pv. *aesculi* strains and plasmid borne expression of GFP appeared adequate for visualization by fluorescence microscopy. The expression of GFP did not significantly alter growth rates when strains were grown in LB (Fig. S1). The plasmid was stably maintained for at least 30 generations when subculturing in medium without antibiotic selection pressure, as was reported for this plasmid when maintained in other hosts [18], [21]. When the *P. s.* pv. *aesculi* PD4818-pMP4655 transformant was inoculated on the stems of *A. hippocastanum* seedlings, typical, elongated necrotic lesions were formed underneath the periderm (Fig. 3A), mainly in the cambium and phloem (Fig. 3B). Given the high plasmid stability and the retained virulence towards *A. hippocastanum* the GFP-expressing transformants were considered useful to enable accurate re-isolation and further studies on bacterial localization and behaviour *in situ*.

### *P. syringae* pv. *aesculi* is Embedded in an Extracellular Matrix *in planta*

Lesions formed by GFP-expressing PD4818 were analysed 4 and 6 weeks after inoculation. The vertical expansion of an infection was sometimes observed as a parabolic shaped row of cells exhibiting elevated cell wall fluorescence, containing a cluster of small, densely packed bacteria in the tip (Fig. 3C/D). Especially the borders of necrotic tissue housed many bacteria. Clustered bacteria occupied empty plant cells (Fig. 3E) and large aggregates of bacteria were found in voids within the necrotic host tissue (Fig. 3F). Individual bacteria in these aggregates were spaced at some distance from their neighbours and did not seem to undergo any type of motion, including Brownian (Fig. 3G). A similar distribution pattern was also observed in lesions formed by wild-type bacteria using immunolabelling (Fig. 3H). The spatial arrangement and motionlessness of these bacteria suggests that formation of a mucoid matrix occurs within host tissues, which might serve a role in the infection and/or survival strategy of the bacteria.

**Figure 4:**
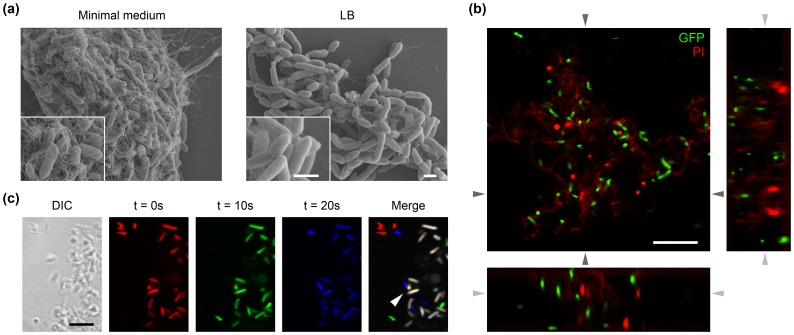
A fibrillar matrix produced *in vitro* by *P. syringae* pv. *aesculi* enwraps aggregates of immobilized bacteria. (A) After incubation in minimal medium a fibrillar material covering clustered bacterial cells is observed by scanning electron microscopy (left-hand panel). Such material is not encountered after incubation in LB, wherein only the polar flagella are observed extracellularly (right-hand panel). The insets show the bacterial cells and the surrounding material in more detail. Note that due to the completely dehydrated state of the specimen, the here presented image likely does not depict the true spatial lay-out of a bacterial cluster. The scale bars indicate 1 µm. (B) A typical cluster observed in a PD4818-pMP4655 culture grown in minimal medium visualized by confocal laser scanning microscopy. GFP-expressing bacteria appear in green, while addition of propidium iodide (PI) brightly stained dead individuals red and led to a mild staining of the amorphous material enwrapping the bacteria within a cluster. The bottom and right panels are computed images of the two orthogonal planes along the z-axis indicated by the dark grey arrows. The light grey arrows indicate the position of the optical plane shown in the top left panel. Note that single bacteria are spaced from neighbouring cells in 3 dimensions. The scale bars indicate 10 µm. (C) Three 10 second interval frames taken from a time-lapse movie of the edge of a PD4818-pMP4655 cluster (depicted in the DIC image) were differentially colour coded and merged to reveal displacement of individual bacteria over time. Several motile bacteria freely moved on the left appearing in a single colour in the merged image, while bacteria within the cluster remained at a fixed location as revealed by their white colour in the merged image. Additionally, oscillatory motion of 2 bacteria is discernable in the cluster margin (arrowhead), indicated by the slight colour shift. The scale bar indicates 5 µm.

To explore whether the matrix is of bacterial origin, *P. s.* pv. *aesculi* PD4818 was grown axenically in a liquid minimal medium. When cultures grown under these conditions were analysed by scanning electron microscopy, cell clusters wrapped in a fibrillar matrix were observed, which was not encountered when cultures were grown under nutrient-rich conditions (Fig. 4A). Repetition of these experiments using the GFP-expressing derivative of strain PD4818 indeed revealed the presence of aggregates of bacterial cells up to approximately 100 µm in size among a population of motile bacteria. Clusters contained both live and dead bacteria. Analysis of optical sections and the orthogonal planes along the z-axis revealed the 3D spacing of bacteria (Fig. 4B). Cells inside a cluster were completely static, although some individuals at the cluster margin sometimes exhibited oscillatory motion (Fig. 4C). These findings support that a bacterium derived matrix could indeed enwrap the individuals within the observed bacterial aggregates *in planta*.

To identify what type of exopolysaccharide could potentially contribute to the observed matrix, *P. s.* pv. *aesculi* was grown in liquid minimal medium with additional salt stress for 2 days. An *m-*hydroxydiphenyl assay revealed the presence of uronic acids in the exopolysaccharide obtained after salt stress (data not shown). High Performance Anion Exchange Chromatography (HPAEC) was used for further identification of the sugar composition of the exopolysaccharide. Under stress conditions the typical degradation products of alginate (guluronic acid and mannuronic acid) were observed as two neighbouring peaks (Fig. S2), although the relative presence of guluronic acid is lower than observed for the alginate derived from brown algae used as control. Taking into account the presence of these two peaks it is concluded that alginate was the predominant polysaccharide in the extracellular material produced by *P. s.* pv. *aesculi* under salt stress. Previous studies point to the possible additional presence of glucuronic acid [22] which elutes approximately at the same time as mannuronic acid. In addition, rhamnose and glucose, although at lower concentrations than alginate, were identified (Fig. S2). Minor traces of an unidentified sugar were also present.

Alginate is often found to be produced by plant pathogenic *Pseudomonas* spp. when associated with their host [23]–[25]. Its secretion aids in retaining water and is thereby thought to contribute to bacterial colonization and symptom development [26]. The ability of *P. s.* pv. *aesculi* to produce alginate was further supported by the presence of genes associated with its biosynthesis and regulation thereof as screened *in silico*. Sequence data of *P. s.* pv. *aesculi* strain 2250 [8] indicated that this strain has a full repertoire of genes encoding alginate biosynthesis and regulatory proteins with close homology to these of *P. s.* pv. *tomato* DC3000 (Table S1). Furthermore, most of the genes predicted to function in biosynthesis were found to be arranged in the canonical alginate biosynthesis operon structure [27].

### Heat-treatment as a Feasible Control Method for Horse Chestnut Bleeding Disease

Once established, bacterial cankers on trees are notoriously difficult to control, largely due to the site of bacterial proliferation that is poorly accessible from the outside [28]. As a method to inactivate the horse chestnut pathogen during its endophytic lifestyle, it was hypothesized that high-temperature treatment may prove adequate. Initial experiments using liquid cultures of *P. s.* pv. *aesculi* pointed out that a 24 hour incubation at 39°C consistently yielded no culturable bacteria (data not shown). Then, to assess the feasibility of such a heat-treatment method *in situ*, the survival of *P. s.* pv. *aesculi* PD4818-pMP4655 was monitored after infected plants were incubated at 39°C for 48 hours. From infected areas of heat-treated plants no viable bacteria were re-isolated, while all untreated lesions readily yielded *P. s.* pv. *aesculi* PD4818-pMP4655 (Table 2). The heat-treated plants showed no visible injury, except occasional damage of leaf edges. These observations indicate that a 2 day incubation at 39°C is sufficient to inactivate bacteria associated with active lesions, while minimally affecting the host plant, thus providing proof of concept for heat-treatment as a high-potential control method to stop horse chestnut bleeding disease progression.

**Table 2:** Effect of heat-treatment on recovery of bacteria from lesions in inoculated *A. hippocastanum* saplings.

Inoculation	Control (no treatment)^a^	39°C for 48 h^a^
PD4818-pMP4655	(11/11)	(0/14)
Mock (PBS)	(0/5)	(0/4)

a The figures are given as number of successful re-isolations per no. of lesions assessed.

## Discussion

The recent emergence of *P. s.* pv. *aesculi* as a pathogen that infects trunks and branches of *Aesculus* spp. has severely affected the vitality of the population of these trees, which are highly regarded for their ornamental value. Although genomic data has shed some light on the origins of pathogenesis, the histopathology of this interaction remained unknown. In this work the histological events after host entry of *P. s.* pv. *aesculi* and its behaviour *in planta* were investigated. Histological responses upon bark injury and bacterial dispersal and clustering were analysed.

The histochemically observed lining of cells with lignified and suberized cell walls in *A. hippocastanum* bark has been described for various other tree species [13]–[16]. Also, in agreement with these earlier reports, the time window in which it is detected in horse chestnut is in the order of several days. However, direct comparison of the speed of its construction is difficult due to a strong correlation with environmental temperature [29].

Both in presence and absence of *P. s.* pv *aesculi*, the wounded bark tissue showed lignification and suberization of the parenchyma cells bordering a wound to a similar extent, suggesting this process is part of an intrinsic defence response of *A. hippocastanum*. However, subtle differences may be present as is reported for some plants in the presence of fungal pathogens [17], [30]. Nevertheless, the observed response is presumably broadly defensive [31], such that a possible specific relation to pathogen presence is obscured.

When monitoring bacterial ingress, it appeared that the lignified and suberized barrier was insufficient or constructed too late to shield off underlying healthy tissue (Fig. 2A). Remarkably, the barrier sometimes seemed a bit fainter near invading bacteria, hinting at pathogen-mediated degeneration (Fig. 2A). This could be facilitated by the various pathways for degradation of lignin-related compounds uncovered in the *P. s.* pv *aesculi* genome [8]. Although not determinative in restraining *P. s.* pv. *aesculi* invasion, presence of the observed barrier might still serve a beneficial, protective role. For instance, it could prevent water and mineral flow towards the bacteria, prevent diffusion of bacterial toxins and effectors to healthy plant tissues, reduce tissue digestibility and give rigidity, preventing further tissue collapse.

Rather than systemic spread through vascular elements, advance of the bacterial infection mostly took place in parenchyma tissue of the bark and presumably through intercellular space (Fig. 2L, M). Under the assumption that this type of tissue is less conductive for bacterial spread, this observation can explain the relatively slow rate of lesion expansion, which is measured to be in the order of 100 µm/day [1] until 1000 µm/day (Our unpublished data). Along with spread throughout parenchyma, bacteria were often found on sclerenchyma fibres (Fig. 2C, N). While the localization and longitudinal orientation of these elements coincides with the site of lesion expansion (Fig. 3B), no function in pathogenicity or bacterial spread could be unequivocally assigned to this apparent association. However, the development of periderm around infected phloem fibres (Fig. 2P) constrains lateral rather than longitudinal spread, potentially explaining the elongated nature of lesions. With the conductive tissue for lesion expansion appearing to be bark parenchyma and/or sclerenchyma, it will be interesting to uncover the means by which the bacterial cells migrate through these tissues. Of particular interest is the potential function of intercellular space in bacterial spread. In mature trees, besides the elongated lesions, laterally spreading lesions are observed as well [11], suggesting different modes of colonization can prevail in individual infections.

The bacterial cells found near the forefront of expanding infections were small and densely clustered (Fig. 3D). Similarly, disease symptoms on the cellular level included occasional colonization of single phloem/cortex parenchyma cells by a large bacterial cluster (Fig. 2B, J). These local bacterial accumulations might be important in the infection process, since expression of pathogenic traits often requires a certain, quorum sensing coordinated, cell density [32]. That such a quorum sensing system may act within the observed bacterial clusters in horse chestnut tissues is supported by the quorum size described for *P. syringae* (down to ∼20 individuals [33]) and the ability of Indian *P. s.* pv. *aesculi* to produce *N*-acyl-homoserine lactones [34].

In later disease stages (i. e. several weeks after infection) bacteria were still found in necrotic tissues (Fig. 2Q–T; Fig. 3E, F). The *ex planta* persistence of bacteria in disrupted tissue might aid spread and reinfection later in time. Individuals were non-motile and spaced at some distance from each other, indicative of a mucoid gel wherein the bacteria were contained (Fig. 3G, H). Similar observations were done on *P. savastanoi* pv. *savastanoi* during its endophytic lifestyle in olive stems [35]. Such a gel may aid the bacterium to create a stress tolerant microenvironment within the host, for example enhancing resistance to dehydration and toxic compounds [26], [36], [37]. Similarly, it may also contribute to bacterial survival on outer tissues [38], [39]. High epiphytical fitness might lengthen the window of opportunity for *P. s.* pv. *aesculi* to gain access to host tissues.

Besides contributing to the resistance to environmental stress, the observed matrix may play a direct role in pathogenicity. One such function may be the suppression of host defence signalling through Ca^2+^ chelation [40]. Another potential mechanism through which a mucoid gel can contribute to virulence has been described for fire blight of pear caused by *Erwinia amylovora*, where the extracellular matrix is hypothesized to drive lesion expansion [41], [42]. Under a rising water potential the matrix can exert a pressure on host tissues, which might even facilitate breaching of defensive barriers constructed by the host plant [41]. If a similar mechanism operates during *P. s.* pv. *aesculi* infection, such pushing forces may also in part explain the seasonal exudate formation typically associated with active lesions (Fig. 1A).

Scanning electron microscopy revealed that the intervening space within an axenically grown bacterial cluster consists of fibrillar material (Fig. 4A). Extracellular polymeric substances produced by *P. syringae* strains include levan and alginate [22], [24], [26], [43]. Our results demonstrate the presence of mannuronic and guluronic acid after hydrolysis of extracellular polysaccharides produced by cell cultures under salt stress. Alginate is thus likely a component of the extracellular matrix, as is found for many phytopathogenic pseudomonads [23], [25]. While isolates of *P. s.* pv. *aesculi* have been reported positive for levan production [9], the HPAEC results of hydrolysed exopolysaccharides in our study did not show the presence of fructose so excluding the production of levan under the investigated conditions. The presence of glucose, rhamnose and glucuronic acid together with alginates in the extracellular matrix has been reported for *P*. *syringae* pv. *syringae* [22] and is in agreement with our results. The capability of *P. s.* pv. *aesculi* to produce alginate is supported by a full complement of biosynthetic and regulatory genes (Table S1). Additionally, uncharacterised components may contribute to the matrix embedding the bacterial cells [43]. Also, factors that coordinate its production require further attention.

The occurrence of matrix embedded bacteria within the host tissues combined with lesion expansion that takes place mostly underneath the periderm (Fig. 3B), imply that once endophytically established, the pathogen is poorly accessible from the outside. This could limit the effectiveness of disease control by agents that require physical contact. Indeed, established cankers in fruit trees caused by *P. syringae* appear notoriously difficult to control [28]. Thus, for management of *P. s.* pv. *aesculi* it may be relevant to target other parts of the (largely unknown) disease cycle or establish measures that can penetrate to reach internal bacterial populations. To this end, a heat-based control method was conceived and tested on *A. hippocastanum* saplings.

Pioneering experiments to test the efficacy of heat-treatment of whole plants indicated that *P. s.* pv. *aesculi* associated with active lesions is rendered inactive after a two day exposure to 39°C, which is tolerated by *A. hippocastanum*. Hence, thermal treatment may be a feasible, non-invasive control method employable for disease management on a larger scale. A potential issue with applying heat-treatment under field conditions could lie in differences in temperature sensitivity that may occur among various isolates, as is reported for *Erwinia amylovora* [44]. However, the extremely high genetic homogeneity among UK *P. s.* pv. *aesculi* isolates [8] implies that variation in heat-resistance among bacterial strains is minimal, suggesting heat-treatment of *A. hippocastanum* may be a widely applicable method.

To date, heat-based phytopathogen control has proven to be a useful method to manage various post-harvest diseases [45], [46]. The results presented in this study demonstrate that heat-treatment can also be used on growing, vegetative plant organs as a control strategy to inactivate *P. s.* pv. *aesculi*. Such treatment could be practiced on seeds, saplings and affected aerial organs of trees, as will be tested in future studies. For mature trees complicating factors like secondary infection by opportunistic pathogens or reinfection should, however, be considered. Nonetheless, heat-treatment could well shift the balance in plant pathogen interaction in favour of the host, enabling sufficient recovery and revitalization in an otherwise lethal infection. In addition, the high potential for repelling this particular disease might mean that this strategy can be expanded to control similar diseases in other tree species caused by related bacterial pathogens.

## Materials and Methods

### Bacterial Strains, Culture Conditions and Plasmids

*Pseudomonas syringae* pv. *aesculi* strains (listed in table 1) were grown in Lysogeny Broth (LB; [47]) at 28°C and 180 rpm. *E. coli* strains (Table 1) were routinely cultured in LB at 37°C and 225 rpm. Plasmid pMP4655 (obtained from the Department of Molecular Microbiology, Leiden University; Table 1) was introduced in *E.coli* DH5α and GM119 by electroporation. Antibiotics were added in the following concentrations unless stated otherwise: tetracycline, 40 µg/mL; carbenicillin, 100 µg/mL and kanamycin, 50 µg/mL. Strains were stored at −80°C for long-term conservation.

### Transformation of *P. s.* pv. *aesculi* by Triparental Conjugation

The two *E. coli* donor strains harbouring pMP4655, helper strain cel40 containing pRK2013 and recipient *P. s.* pv. *aesculi* strains were grown until late log phase. Antibiotics present in the initial culture medium were first removed by collecting cells at 1,000× *g* for 5 min and resuspending them in their original culture volume of LB. All cultures were pelleted again and resuspended in 3% of their original culture volume of LB. Aliquots of 30 µL were mixed in a 1∶1:2.5 and 1∶1:5 (helper:donor:recipient) ratio and transferred to sterile, 20 mm ø BA85 nitrocellulose filters with 0.45 µm pore size (Schleicher & Schuell), which were incubated overnight on LB plates. The cells were collected from the filters in 1 mL PBS and plated on LB plates containing carbenicillin and 15 µg/mL tetracycline. Well grown colonies were serially diluted in PBS and restreaked on plates containing carbenicillin and 30 µg/mL tetracycline to yield pure *P. s.* pv. *aesculi* transformants. Both *E. coli* donorstrains appeared equally potent for conjugal transfer of pMP4655.

### Plant Cultivation and Inoculation

For the analysis of the early defence response and bacterial invasion upon inoculation, experiments were conducted with one and two-year old horse chestnut (*A*. *hippocastanum*) seedlings. Plants were maintained at room temperature under natural light in containers with 5 L potting soil and watered weekly. Experiments were repeated three times. Eight plants in total were inoculated at various sites on the stem for sampling in time, two inoculations at opposite sides of the stem. At each sample moment (see ‘*Sampling, fixation and sectioning*’) one set of opposite inoculation sites was removed. Plants were either mock-inoculated as a control (3 plants in total) or inoculated with *P. s.* pv. *aesculi* (5 plants in total). For artificial infection of the plants a superficial wound was inflicted on a surface sterilized part of the stem by making a small (2–3 mm), diagonal cut with a downward angle using a sterile razorblade. Care was taken not to cut deeper than the cambial zone. These wounds were inoculated by pipetting 5 µL of sterile PBS or 5 µL PBS containing 10^8^ cfu/mL *P. s.* pv. *aesculi* PD4818 into the wound fissure.

For the analysis of bacterial spread on the long term, 20 plants were inoculated at 6 sites per plant (including 5 mock inoculated plants). At each sample moment 4 plants were harvested (one of them mock inoculated) and of each plant one sample was selected randomly for analysis.

Infection experiments with GFP expressing bacteria (isolate PD4818 harbouring pMP4655) were performed on three separate occasions in climate cells using 6 two-year old horse chestnut seedlings maintained at 21°C, 70–90% RH and a 16 h light, 8 h dark cycle. Plants were sampled after 4 and 6 weeks to examine *in planta* behaviour of bacteria, and monitored over a period of 8 weeks.

### Sampling, Fixation and Sectioning

For the analysis of the early stages of the infection process samples of stem were removed from the inoculated seedlings around the original inoculation site at 0, 1, 2, 3, 4, 6, 8 and 14 days after inoculation. For investigation of later stages, samples were taken at 2, 3, 4, 10 and 18 weeks after inoculation. The sampled pieces of stem were fixed in 4% w/v paraformaldehyde, 0.05% v/v glutaraldehyde and 0.01% v/v Triton X-100 in 0.1 M phosphate buffer (pH 7.2) by vacuum infiltration (10 kPa). Infection experiments with GFP-expressing bacteria were sampled at 4 and 6 weeks after inoculation and two inoculation sites were examined per time-point. When preparing samples with GFP-expressing bacteria, the fixation step was omitted. Transverse and longitudinal sections of ∼20 µm thick were made with a Reichert sledge microtome. Fixed samples were stored in 0.1 M phosphate buffer (pH 7.2) containing 0.02% w/v NaN_3_ at 4°C.

### Staining and Immunolabelling

To stain lignin, sections were incubated in 10% w/v phloroglucinol in ethanol for 20 min and subsequently in 20% v/v HCl for 3 min. Sections were subsequently washed twice for 30 seconds in dH_2_O and mounted in glycerol. To stain fats and waxes, sections were incubated in 1% w/v Sudan IV for 10 min, rinsed in 50% v/v ethanol for 1 min and mounted on a slide in glycerol. Wild-type *P. s.* pv. *aesculi* was visualized using FITC conjugated anti-PRI1 antibodies (PRI, Wageningen, The Netherlands). Sections were washed in PBS for 10 min, incubated in 0.1 M hydroxyl-ammoniumchloride for 30 min and washed again in PBS for 5 min. Sections were blocked in 0.5% w/v Bovine Serum Albumin Fraction V (Sigma-Aldrich) for 30 min and rinsed twice with PBS for 15 min. Samples were incubated overnight at 4°C in anti-PRI1-FITC diluted 1∶600 or 1∶1200 in PBS, washed twice for 15 min and twice for 30 min in PBS. The sections were finally mounted on slides in glycerol Citifluor AF2 for microscopic observation. For vitality assessments liquid grown bacteria were stained with propidium iodide (Sigma) at a final concentration of 5 µM for 5 min in the dark.

### Image Acquisition and Processing

Slides with anti-PRI1-FITC and GFP labelled bacteria were analysed with a Nikon Optiphot microscope under bright field and fluorescence conditions. For fluorescence a 100 W high pressure HB-10101AF mercury lamp (Nikon) and FITC filters were used. Images were acquired with a Kappa DX-20 camera. For smaller magnifications a Zeiss Discovery V12 stereomicroscope equipped with a JENOPTIK ProgRes C10+ camera was used. Bacterial clusters grown axenically were observed with a Zeiss Axiovert 200 M microscope connected to a Zeiss LSM510 META confocal scanning system using a 63× PlanApochromat 1.4 N.A. oil immersion objective. All images were processed with ImageJ 1.43 (http://rsbweb.nih.gov/ij/) and Photoshop 8 CS (Adobe systems Inc.).

### In vitro Production of Extracellular Matrix and Scanning Electron Microscopy

To observe the production of extracellular material, *P. s.* pv. *aesculi* PD4818 and its pMP4655 harbouring derivative were cultured at 20°C in either LB or minimal medium containing 20 mM NaCl, 20 mM phosphate buffer, 10 mM (NH_4_)_2_ SO_4_, 5 mM MgSO_4_, 10 mM fructose and 10 mM mannitol (pH 6.1) [50], [51]. After 2–3 days, cultures were observed by fluorescence microscopy as described above or prepared for scanning electron microscopy by fixing 1 mL samples with 3% v/v glutaraldehyde dissolved in the appropriate medium. Droplets of the sample were brought on Poly-L-lysine hydrobromide (Sigma) coated, circular cover slips (Menzel). After rinsing with water, the samples were dehydrated in series of acetone solutions and subsequently critical point dried with carbon dioxide using a BalTec CPD 030. Samples were sputter coated with 5 nm platinum in a dedicated preparation chamber (CT 1500 HF, Oxford Instruments) and analysed with a JEOL 6300F field emission scanning electron microscope. Images were digitally recorded using an Orion 6 PCI interface (Eli SPRL) and processed with Adobe Photoshop 8 CS.

### Characterisation of Extracellular Polysaccharides

Cultures of 125 mL of *P. s.* pv. *aesculi* PD4818 were either grown in duplo in minimal medium for 5 days or in minimal medium for 3 days and then under salt stress (0.4 M NaCl in minimal medium) for 2 days. Cultures were centrifuged for 15 min (10,000×*g*, RT) and three volumes of ice cold ethanol were added to the supernatant to precipitate the extracellular material o/n at 4°C. The precipitate was collected after centrifugation (30 min, 10,000×*g*, 4°C) and subsequently dialyzed o/n (Medicell Visking, MWCO 12,000–14,000 Da) against dH_2_O. Four volumes of ice cold acetone were added to the retentate and after o/n precipitation at 4°C the extracellular material was collected by centrifugation for 30 min at 10,000×*g* (10°C). The solid material was washed with acetone and dried at 35°C. Commercial alginates, sodium alginate from *Laminaria hyperborea* (BDH Chemicals) and *Macrocystis pyrifera* (Sigma-Aldrich), and the dried extracellular material were hydrolysed in 0.375 ml 72% w/v H_2_SO_4_ for 30 min at 30°C, continued in 1 M H_2_SO_4_ for 3 h at 100°C. The amount of uronic acids was determined using the *m*-hydroxydiphenyl assay as described by Blumenkrantz and Asboe-Hansen [52] and galacturonic acid was used as standard.

The sugar composition was determined in more detail by High Performance Anion Exchange Chromatography (HPAEC) using an ICS-3000 Ion Chromatography HPLC system equipped with a CarboPac PA-1 column (2×250 mm) in combination with a CarboPac PA guard column (2×25 mm) and a pulsed electrochemical detector in pulsed amperometric detection mode (Dionex). A flow rate of 0.3 mL min^−1^ was used and the column was equilibrated with 16 mM NaOH. The following gradient was used: 0–26 min, 16 mM NaOH; 26–33 min, 16–100 mM NaOH; 33–78 min 0–1 M sodium acetate in 100 mM NaOH; 78–83 min 1M sodium acetate in 100 mM NaOH. Before analysis samples were diluted in water and 2.5 µL/mL 0.1% w/v bromine phenol blue in ethanol was added to the samples. To adjust the pH, solid sodium carbonate was added until a clear magenta colour was obtained. Subsequently the solution was filtered through a 0.45 µm PTFE filter. Rhamnose, fucose, mannitol, arabinose, glucose, xylose, galactose, sucrose, glucuronic acid and galacturonic acid (Sigma Aldrich) were used as standards for identification.

### Heat-treatment

To test the efficacy of thermal treatment on active lesions, eight 4 year old *A. hippocastanum* saplings were inoculated with *P. s.* pv. *aesculi* PD4818-pMP4655 and two plants were inoculated with PBS. The saplings were maintained in climate cells as described above. Two months after inoculation half of the population was covered with plastic and incubated in a climate cell heated to 39°C for 48 h. Hereafter the plants were again incubated under the standard conditions. One day after treatment, expanded lesions were removed from all sapling stems, always excluding the original inoculation site (except for mock-inoculations). Samples were homogenized and put in an amount of PBS proportional to the weight of the sampled tissue. The equivalent of 0.5 mg tissue and a series of seven subsequent 10-fold dilutions were then spotted onto carbenicillin containing LB plates. Plates were incubated at 20°C for 4 days and then scored for bacterial growth. The identity of isolated bacteria was confirmed by fluorescence microscopy. Plant growth and appearance was monitored after heat-treatment for 2 months.

## Supporting Information

Figure S1 Growth of two *Pseudomonas syringae* pv. *aesculi* strains and their GFP-expressing derivatives in LB.
(PDF)

Figure S2 High performance anion exchange chromatography of acid hydrolysed extracellular polysaccharide of *Pseudomonas syringae* pv. *aesculi* PD4818.
(PDF)

Table S1 List of predicted homologues of alginate biosynthesis and regulatory proteins in *Pseudomonas syringae* pv. *aesculi* 2250.
(DOC)
