# Supplementary material for: Histological Examination of Horse Chestnut Infection by Pseudomonas syringae pv. aesculi and Non-Destructive Heat Treatment to Stop Disease Progression
Source: PLoS One. 2012 Jul 9;7(7):e39604. doi: 10.1371/journal.pone.0039604 (PMC3392261; doi:10.1371/journal.pone.0039604)
Supplement: Figure S1 — Growth of two Pseudomonas syringae pv. aesculi strains and their GFP-expressing derivatives in LB. (PDF) [file pone.0039604.s001.pdf]

Supplemental figure S1

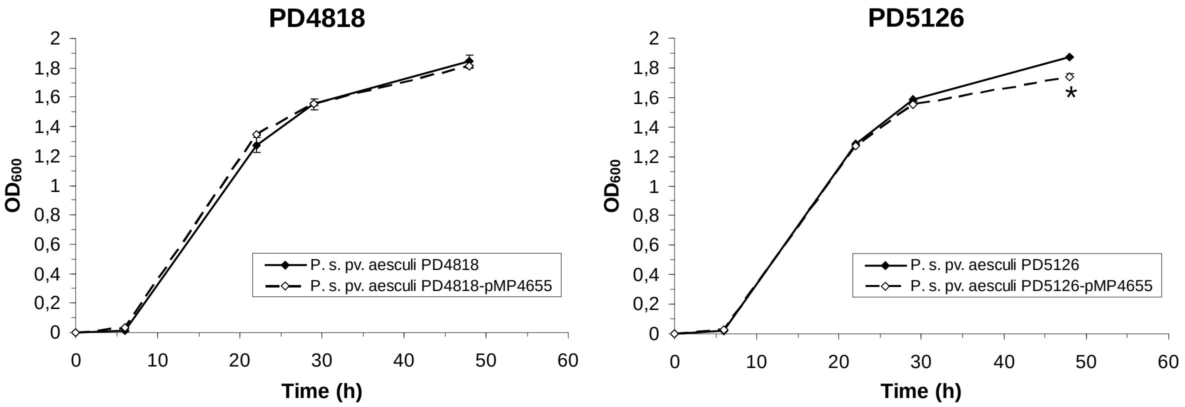

Growth of two *Pseudomonas syringae* pv. *aesculi* strains and their GFP-expressing derivatives. Overnight cultures of *P. s.* pv. *aesculi* PD4818, PD5126 and their derived transformants harbouring pMP4655 were diluted to an OD_{600} of 0.005 in fresh LB. The optical density at 600 nm of the cultures was recorded during growth at 28 °C and 180 rpm. The presented values are means (± standard deviation) of three replicates and an asterisk indicates significant differences as determined with Students 2-tailed *t*-test (P<0.001).
