# Supplementary material for: Histological Examination of Horse Chestnut Infection by Pseudomonas syringae pv. aesculi and Non-Destructive Heat Treatment to Stop Disease Progression
Source: PLoS One. 2012 Jul 9;7(7):e39604. doi: 10.1371/journal.pone.0039604 (PMC3392261; doi:10.1371/journal.pone.0039604)
Supplement: Figure S2 — High performance anion exchange chromatography of acid hydrolysed extracellular polysaccharide of Pseudomonas syringae pv. aesculi PD4818. (PDF) [file pone.0039604.s002.pdf]

Supplemental figure S2

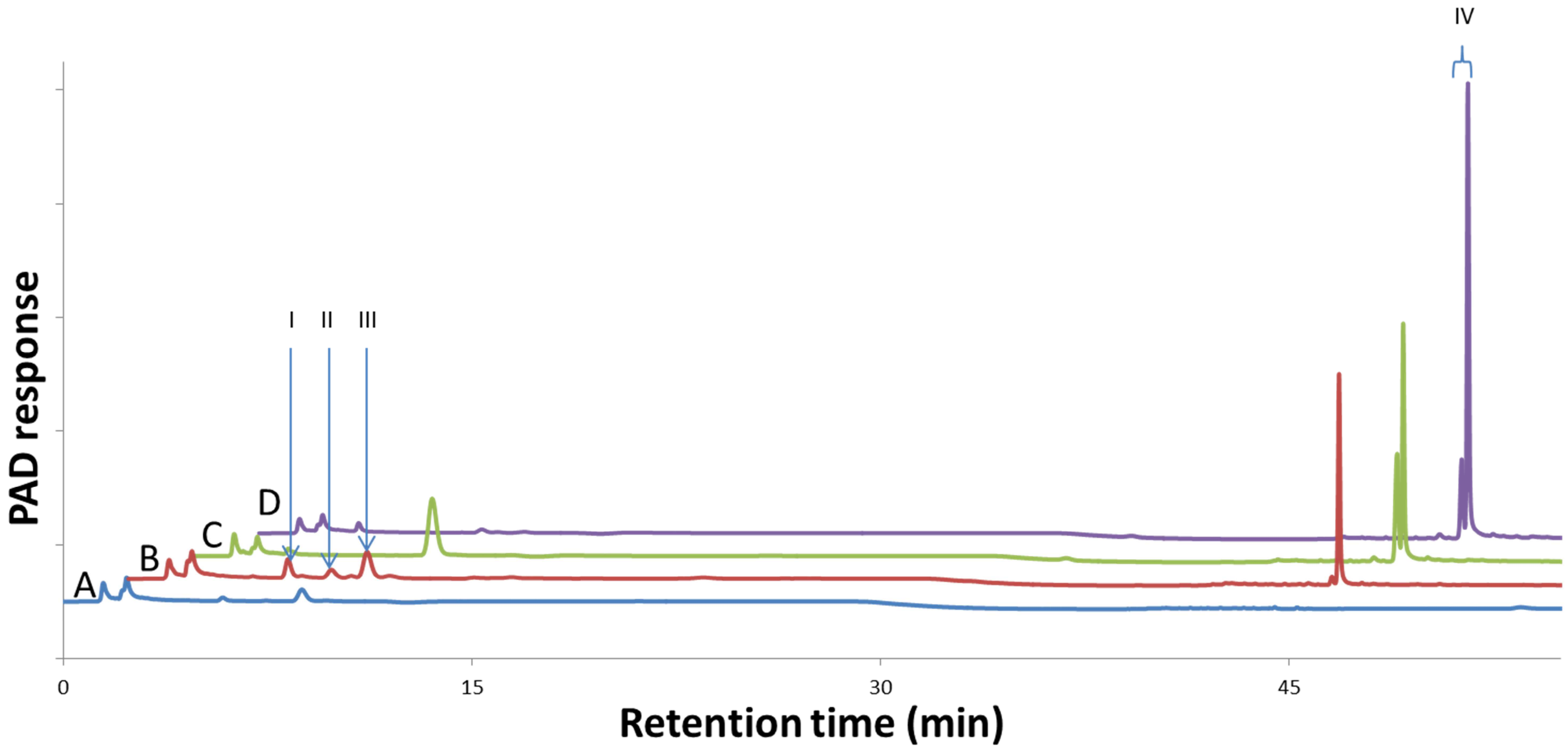

High performance anion exchange chromatography of acid hydrolysed extracellular polysaccharide of *Pseudomonas syringae* pv. *aesculi* PD4818 cultivated without (**A**) and with salt stress (**B**) and acid hydrolysed alginate from *Laminaria hyperborea* (**C**) and *Macrocystis pyrifera* (**D**). Each subsequent plot has been shifted 2.4 minutes for clarity. The marked peaks represent: **I**, Rhamnose; **II**, Unidentified compound; **III**, Glucose; **IV**, Degradation pattern of alginate (guluronic acid and mannuronic acid).
