# Supplementary material for: Histological Examination of Horse Chestnut Infection by Pseudomonas syringae pv. aesculi and Non-Destructive Heat Treatment to Stop Disease Progression
Source: PLoS One. 2012 Jul 9;7(7):e39604. doi: 10.1371/journal.pone.0039604 (PMC3392261; doi:10.1371/journal.pone.0039604)
Supplement: Table S1 — List of predicted homologues of alginate biosynthesis and regulatory proteins in Pseudomonas syringae pv. aesculi 2250. (DOC) [file pone.0039604.s003.doc]

| **Table S1**. Predicted homologues of alginate biosynthesis and regulatory proteins in *Pseudomonas syringae pv. aesculi* | | | | |
| --- | --- | --- | --- | --- |
| Alginate protein | Description | *P. syringae pv. aesculi* 2250 accession no. | Predicted size (a. a.) | % identity^a^ |
| AlgC | alginate biosynthesis protein AlgC | ZP_06477709 | 465 | 96% |
| AlgD | GDP-mannose 6-dehydrogenase | ZP_06478555 | 438 | 98% |
| Alg8 | alginate biosynthesis protein Alg8 | ZP_06478556 | 493 | 97% |
| Alg44 | alginate biosynthesis protein Alg44 | ZP_06478557 | 390 | 92% |
| AlgK | alginate biosynthesis protein AlgK | ZP_06478558 | 470 | 94% |
| AlgE | alginate biosynthesis protein AlgE | ZP_06478559 | 493 | 97% |
| AlgG | alginate biosynthesis protein AlgG | ZP_06478560 | 536 | 92% |
| AlgX | alginate biosynthesis protein AlgX | ZP_06478561 | 479 | 97% |
| AlgL | poly(beta-D-mannuronate) lyase | ZP_06478562 | 378 | 96% |
| AlgI | alginate biosynthesis protein AlgI | ZP_06478563 | 518 | 97% |
| AlgJ | alginate biosynthesis protein AlgJ | ZP_06478564 | 391 | 95% |
| AlgF | alginate biosynthesis protein AlgF | ZP_06478565 | 222 | 94% |
| AlgA | alginate biosynthesis protein AlgA | ZP_06478566 | 483 | 99% |
|  |  |  |  |  |
| AlgP | alginate regulatory protein AlgP | ZP_06477756 | 341 | 81% |
| AlgQ | anti-RNA polymerase sigma 70 factor | ZP_06477758 | 157 | 96% |
| AlgR | alginate biosynthesis regulatory protein AlgR | ZP_06477764 | 248 | 93% |
| AlgZ | sensor histidine kinase FimS | ZP_06477765 | 360 | 95% |
| AlgB | alginate biosynthesis transcriptional regulatory protein AlgB | ZP_06477794 | 448 | 99% |
| AlgN | sigma E regulatory protein, MucB/RseB | ZP_06481238 | 319 | 87% |
| MucA | alginate regulatory protein MucA | ZP_06481239 | 196 | 99% |
| AlgU (=AlgT) | RNA polymerase sigma factor AlgU | ZP_06481240 | 193 | 99% |

^a^ Percentage amino acid identity when the *P. s.* pv. *aesculi* 2250 sequence was compared to the sequence published for *P. s.* pv. *tomato* DC3000 (GenBank AE016853)
